# The protective mechanism of protein kinase R to inhibit neuronal ferroptosis in cerebral injury from subarachnoid hemorrhage

**DOI:** 10.1002/brb3.2722

**Published:** 2022-07-27

**Authors:** Jianwei Lei, Shuxin Song, Zhihua Chen, Sihong Shu, Qiang Liu, Wei Hu

**Affiliations:** ^1^ Department of Neurosurgery the Second Affiliated Hospital of Nanchang University Nanchang Jiangxi China

**Keywords:** ferroptosis, protein kinase R, RNA sequencing, subarachnoid hemorrhage

## Abstract

**Purpose**: To investigate the role and mechanism of protein kinase R (PKR) in subarachnoid hemorrhage (SAH)‐mediated ferroptosis.

**Methods**: A rat SAH model was constructed and treated with PKR inhibitor C16 to observe SAH and neurological impairment in rats and to detect malonaldehyde (MDA), iron ions content, ferritin heavy polypeptide 1 (FTH1) and glutathione peroxidase 4 (GPX4), and other related ferroptosis indicators in brain tissue. RNA sequencing analysis was used to investigate the mechanism of PKR, affecting the ferroptosis network of SAH.

**Results**: SAH caused severe fundic hemorrhage, neurological impairment, MDA and iron ion accumulation, and significant decrease in GPX4 and FTH1 levels in rats. C16 treatment significantly improved the above signs caused by SAH. By RNA‐seq analysis, brain tissue of SAH‐treated rats with SAH and C16 differentially expressed mRNA target genes enriched in stress response and organic developmental signaling pathways.

**Conclusion**: Inhibition of PKR may improve cerebral injury after SAH by inhibiting ferroptosis, and RNA sequencing staged its mechanism of action may be related to the stress response.

## Graphical Abstract

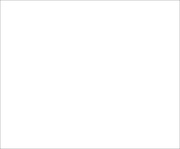

## 1 INTRODUCTION

Subarachnoid hemorrhage (SAH) refers to a clinical syndrome caused by the rupture of a diseased vessel at the base or surface of the brain and the direct flow of blood into the subarachnoid space. SAH is a common critical clinical emergency with a high mortality and disability rate (Sehba et al., 2011). The concept of early brain injury (EBI) refers to pathophysiological changes within 3 days after the onset of the disease, including increased intracranial pressure, disruption of the brain barrier, inflammatory cascade, oxidative stress, and apoptosis. The current study found that EBI is a key adverse prognostic factor for SAH (Fang et al., 2019; Fujii et al., 2013), causing blood–brain barrier disruption, cerebral edema, acute cerebral vasospasm, and microvascular dysfunction. Neuroapoptosis is an important factor in triggering a range of injuries in EBI; yet, drugs targeting neuroapoptosis have not improved the prognosis of patients with SAH (Zhan et al., 2012; Zille et al., 2017).

Ferroptosis is a recently discovered form of programmed cell death characterized by excessive accumulation of lipid reactive oxygen species (ROS), depletion of glutathione (GSH), glutathione peroxidase 4 (GPX4) inhibition, and the accumulation of large amounts of intracellular lipid peroxides, leading to cell death (Cao & Dixon, 2016; Dixon et al., 2012; Xie et al., 2016). Ferroptosis is genetically, biologically, and morphologically distinct from other forms of cell death (apoptosis, necrosis, scorching, etc.), and a growing number of studies have shown that ferroptosis is associated with a variety of human diseases, including cancer, neurodegenerative diseases, ischemic reperfusion injury, and kidney disease (Carbone & Melino, 2019; Derry et al., 2020; Müller et al., 2017; Su et al., 2019).

Ferroptosis is one of the major forms of neuronal death after SAH, which occurs from the release of iron ions from hemoglobin/hemoglobin catabolism after bleeding, and recent studies have confirmed that symptoms of EBI can be effectively alleviated by using iron death pathway inhibitors (Cao et al., 2021; Li et al., 2021; Qu et al., 2021). Thus, clarifying the key role of neuronal ferroptosis in the formation of EBI and effectively inhibiting neuronal ferroptosis during EBI may be a key aspect in the clinical management of SAH.

Protein kinase R (PKR) is a serine‐threonine kinase encoded by the EIF2AK2 gene in humans that can be induced and activated by interferon (IFN). PKR was first cloned in 1990 at the Pasteur Institute and is also known as a double‐stranded RNA‐dependent protein kinase or IFN‐induced double‐stranded RNA‐dependent protein kinase (Feng et al., 1992). PKR plays important cellular processes such as mRNA transcription, transcriptional regulation, apoptosis regulation, and proliferation (Garcia et al., 2007; Taylor et al., 2005). PKR can regulate apoptosis, and it has been shown that when the protein PKR is knocked down, Fas mRNA expression is inhibited, allowing the Fas‐induced apoptotic pathway to be suppressed as well (Gil & Esteban, 2000). Dysregulation of PKR is associated with diseases such as cancer, design degenerative diseases, inflammation, and metabolic disorders (Morel et al., 2009). In addition, PKR is associated with ferroptosis, and the role of PKR is to promote the occurrence of ferroptosis (Hirata et al., 2019). The PKR inhibitor C16 can prevent oxytosis and ferroptosis in HT22 cells by inhibiting the accumulation of ROS (Hirata et al., 2019).

No studies on the mechanism of PKR action in SAH have been reported. This study will explore the role of PKR in ferroptosis in SAH and provide new theoretical support for the development of drugs targeting it. Moreover, this study will broaden the research field of SAH and use RNA‐seq to explore the related differential expression factors to further reveal the specific mechanism of ferroptosis in SAH and the role network of PKR.

## 2 MATERIALS AND METHODS

### 2.1 Animals and groups

Twenty‐four male SD rats, purchased from Beijing Spelford Biotechnology Co., Ltd, were divided into the sham‐operated group (CON), the SAH model group (SAH), and the SAH model + C16 (PKR inhibitor) administration group (SAH + C16).

The SAH model was established using the internal carotid artery puncture method. The rats were anesthetized with intraperitoneal injection of sodium pentobarbital and fixed in the supine position. An oblique incision of approximately 2 cm in length was made on the right side of the midline of the neck to expose and separate the common carotid artery, external carotid artery, and internal carotid artery. The common and internal carotid arteries were clamped with an arterial clamp, and the external carotid artery was ligated with a nylon suture and incised proximal to the ligature. The arterial clamp on the internal carotid artery is removed, and the middle cerebral artery occlusion monofilament is pushed through the external carotid artery into the internal carotid artery, and when a certain resistance is felt, the monofilament is pushed forward another 1–2 mm to perforate the vessel. The SAH model was established in the SAH and SAH + C16 groups, and the CON group was only surgically operated but not perforated. The C16 (MedChemExpress LLC, Cat#HY13977A, Shanghai, China) injected through the left ventricle 1 h before modeling.

### 2.2 Brain tissue injury scoring

The brain base tissue was divided into six sections, and each section was scored as follows: 0 for no blood in the subarachnoid space, 1 for a small amount of blood in the subarachnoid space, 2 for moderate clotting with identifiable arteries, and 3 for a clot covering all arteries. The sum of the six‐part scores was used as the total score for fundic hemorrhage.

The Garcia 18‐point evaluation method was used to score neurological function after 48 h of modeling: six items including voluntary movement, symmetry of limb movement when the rat's tail was held in suspension, observation of forepaw extension when the rat's tail was held on the edge of the table, ability to climb and grasp the cage, body sensory response, and response to touching the whiskers, with a maximum total score of 18 points.

### 2.3 Reverse transcription‐polymerase chain reaction

After 48 h of modeling, the rats were euthanized, and their brain tissues were collected. Total RNA was isolated using TRIzol® reagent (Invitrogen, Thermo Fisher Scientific, Cat# 10296028). Single stranded cDNA was synthesized from 0.5 μg of total RNA with oligo (dT)12‐18 primer (Sangon, Shanghai, China) and SuperScript™ III RNase‐reverse transcriptase kit (Thermo Fisher Scientific, Cat# 11752050). The reactions were incubated for 60 min at 50°C and were then terminated by heating for 15 min at 75°C. cDNA was amplified in Polymerase Chain Reaction (PCR) buffer by denaturing for 30 s at 94°C, annealing for 30 s at 58°C, and extending for 30 s at 72°C. This process was performed for 30 cycles. The PCR products were analyzed on 1.5% or 2% agarose gels (Invitrogen, Thermo Fisher Scientific, Cat# 16500100) containing 0.5 μg/ml ethidium bromide. Images of typical agarose gel electrophoresis were captured using a Gel Image System (UVP, USA, Cat# GelDoc‐It310) and analyzed using a 2^–∆∆Ct^ methods.

### 2.4 Western blot analysis

Twenty micrograms of protein were subjected to Sodium Dodecyl Sulfate PolyAcrylamide Gel Electrophoresis (SDS‐PAGE) (10% or 12%) and electro‐transferred to PVDF membrane (Millipore, USA, Cat# ISEQ00010). After blocking in 5% nonfat dry milk in PBS with 0.05% Tween20, membranes were probed with the following specific antibodies: antiferritin heavy polypeptide 1 (FTH1) antibody (1:1000, Bioss Biotechnology Co. Ltd, Beijing, China, bs‐5907R), anti‐GPX4 antibody (1:1000, Bioss, bs‐3884R), anti‐PKR (1:1000, Beijing Biolab Technology Co., Ltd, China, K22624‐RBE), and antiactin antibody (1:2000, Bioss, bs‐10966R). Quantification of the bands was performed using a ChemiDoc MP chemiluminescence gel imaging system (Bio‐rad, Inc., USA, CAT# 170–8280).

### 2.5 Biochemical indexes analysis

Free iron content, malonaldehyde (MDA), and GPX4 activity in rat brain tissues were measured according to the manufacturer's instructions of the corresponding kits. The iron ion assay kit and MDA assay kit were purchased from Nanjing Jiancheng Biological Company (CAT# A039‐2‐1 and A003‐1). The GPX4 activity assay kit was purchased from Solarbio Life Sciences Company (CAT# BC1195).

### 2.6 RNA sequencing

The sequence libraries were generated using the TruSeq mRNA sample preparation kit from Illumina (San Diego) by use of the Sciclone NGS Liquid Handler (Perkin Elmer) according to the manufacturer's instructions. After extra purification of the libraries, we sequenced 9 pmol of each obtained cDNA fragment library on an Illumina HiSeq2500 High Output Standard using default parameters.

Good quality of the data was ensured by showing on average Phred‐scores exceeding the predefined minimum of 30 in over 90% of the reads of each position, equivalent to a base call accuracy of 99.9%. Quality control of the reads, alignment to the build 37 human reference genome, and subsequent gene annotation are described in detail in the Supporting Information. The average alignment of the reads to the human reference genome (uniquely mapped reads) was 86% (range, 73−90%).

### 2.7 Differential expression analysis

We used the counts per gene of each sample obtained after alignment as input for subsequent differential expression analyses. Counts of each sample were normalized using the trimmed mean of M‐values to remove systematic bias caused by technical variability between libraries. To account for biological variation, we calculated common and tag‐wise dispersion rates using the Cox‐Reid profile‐adjusted likelihood method. Tag‐wise dispersion was shrunken towards the mean to account for overdispersion, for which we set the degree of freedom at 5. We designed a negative binominal generalized log‐linear regression model including age as covariate, which was fitted to the read counts for each gene for genewise statistical testing, followed by a likelihood ratio test. We considered genes with a two‐tailed *p* < .05 after false discovery rate correction according to Benjamini and Hochberg to be differentially expressed. Analyses were performed using the package edgeR (version 3.6.2)13 in the R software (version 3.1.0; http://www.r‐project.org/), the Bioconductor (version 2.14; http://www.bioconductor.org), and the supporting packages limma (version 3.20.2) and biomaRt (version 2.20.0).

### 2.8 Bioinformatics analysis

Gene ontology (GO) functional annotation enrichment analysis of differential proteins: differentially expressed proteins were screened according to the inclusion criteria and compared with all proteins in the known human proteome, that is, GO database logical comparison, to calculate the number of differentially expressed proteins in the important GO classification entries, and then Top GO analysis was applied to find out the GO classification with significant enrichment of candidate proteins. Inclusion criteria were: top 10 GO items with *p*‐value less than .05 and Enrichment Score. DAVID software (https://david.ncifcrf.gov/) was used for GO functional classification and enrichment analysis, and significantly enriched GO terms provided a basis for the study of biological functions of differentially expressed proteins (Liang et al., 2014, 2016; Zhao et al., 2020).

Kyoto Encyclopedia of Genes and Genomes (KEGG) analysis is an important technical method for high‐throughput omics analysis of biological pathways, which can be used to infer the critical signaling pathways in which candidate differential proteins may be involved. The channels with *p* < .05 and ranking top 20 in rich set score were selected. The enriched metabolic pathways and signaling pathways involved were analyzed by comparing the number and degree of difference (*p* value) of the differentially proteins (Liang et al., 2014, 2016; Zhao et al., 2020).

### 2.9 Statistical analysis

The measurement data were expressed as mean ± standard deviation (SD). For continuous variables, independent sample Student's *t*‐test was used for comparison between groups, and *p* < .05 was considered statistically significant. All data were analyzed using SPSS software (Version 22, Chicago, IL, USA).

## 3 RESULTS

### 3.1 Inhibition of PKR expression significantly improved EBI after SAH

**FIGURE 1:**
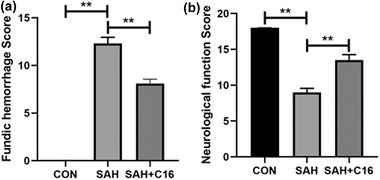
Brain tissue injury score suggested that inhibition of PKR expression could significantly improve EBI after SAH. (a) Fundic hemorrhage score of rats in each group; (b) Garcia neurological function scores of rats in each group; **p < .01

By constructing a rat SAH model and giving C16 treatment, the rats were scored for cerebral floor hemorrhage and neurological function 48 h after modeling. The scoring results are shown in Figure **1**. The rats with SAH model had significantly more cerebral base hemorrhage (*p* < .01) and significantly lower Garcia score compared with the control rats (*p* < 0.01). In contrast, C16 treatment significantly reduced the amount of cerebral base hemorrhage (*p* < 0.01) and effectively reduced the neurological function scores (*p* < .01) of rats compared with the SAH group. These results suggest that PKR inhibition has an ameliorative effect on EBI after SAH in rats.

### 3.2 Inhibition of PKR significantly reduced the increase in ferroptosis indexes caused by SAH

**FIGURE 2:**
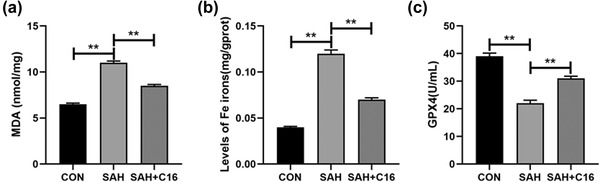
Inhibition of PKR ameliorated the accumulation of MDA and iron ions and the reduced GPX4 activity caused by SAH; (a) MDA levels in the brain tissue of rats in each group; (b) iron ion levels in the brain tissue of rats in each group; (c) GPX4 activities in the brain tissue of rats in each group; **p < .01

Forty‐eight hours after the SAH model was constructed in rats, brain tissues were collected and assayed for MDA and iron ion levels and GPX4 activity using kits. It was found that the indicators of ferroptosis were significantly higher in the SAH model group compared to the CON group: MDA and iron ion levels in brain tissue were significantly higher, while GPX4 activity was significantly lower (*p* < .01, Figure **2**). In contrast, treatment of rats with the PKR inhibitor C16 significantly alleviated the accumulation of MDA and iron ions and the reduction of GPX4 activities induced by SAH (*p* < .01, Figure **2**).

**FIGURE 3:**
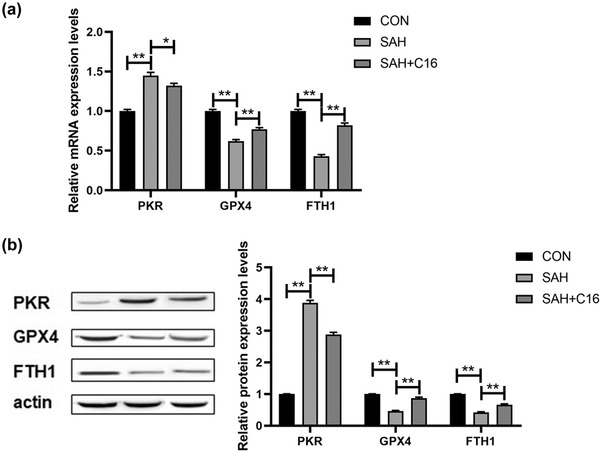
Inhibition of PKR ameliorates the decrease in mRNA and protein expression levels of GPX4 and FTH1 induced by SAH. (a) mRNA expression levels of PKR, GPX4, and FTH1 in rat brain tissues of each group; (b) protein expression levels of PKR, GPX4, and FTH1 in rat brain tissues of each group; **p < .01

In addition, the mRNA and protein levels of PKR in rat brain tissues were examined to verify our animal model design, and SAH significantly increased PKR levels in rat brain tissues, while C16 effectively inhibited PKR expression (*p* < .01, Figure **3**). Besides, SAH significantly decreased the mRNA and protein expression levels of GPX4 and FTH1, in rat brain tissues, while inhibition of PKR significantly increased the expression levels of GPX4 and FTH1 that were decreased by SAH (*p* < .01, Figure **3**). Thus, SAH caused a significant increase in PKR levels in rat brain tissue, and inhibition of PKR could effectively improve the elevated ferroptosis indexes caused by SAH.

### 3.3 Analysis of RNA sequencing results of rats’ brain tissue

**FIGURE 4:**
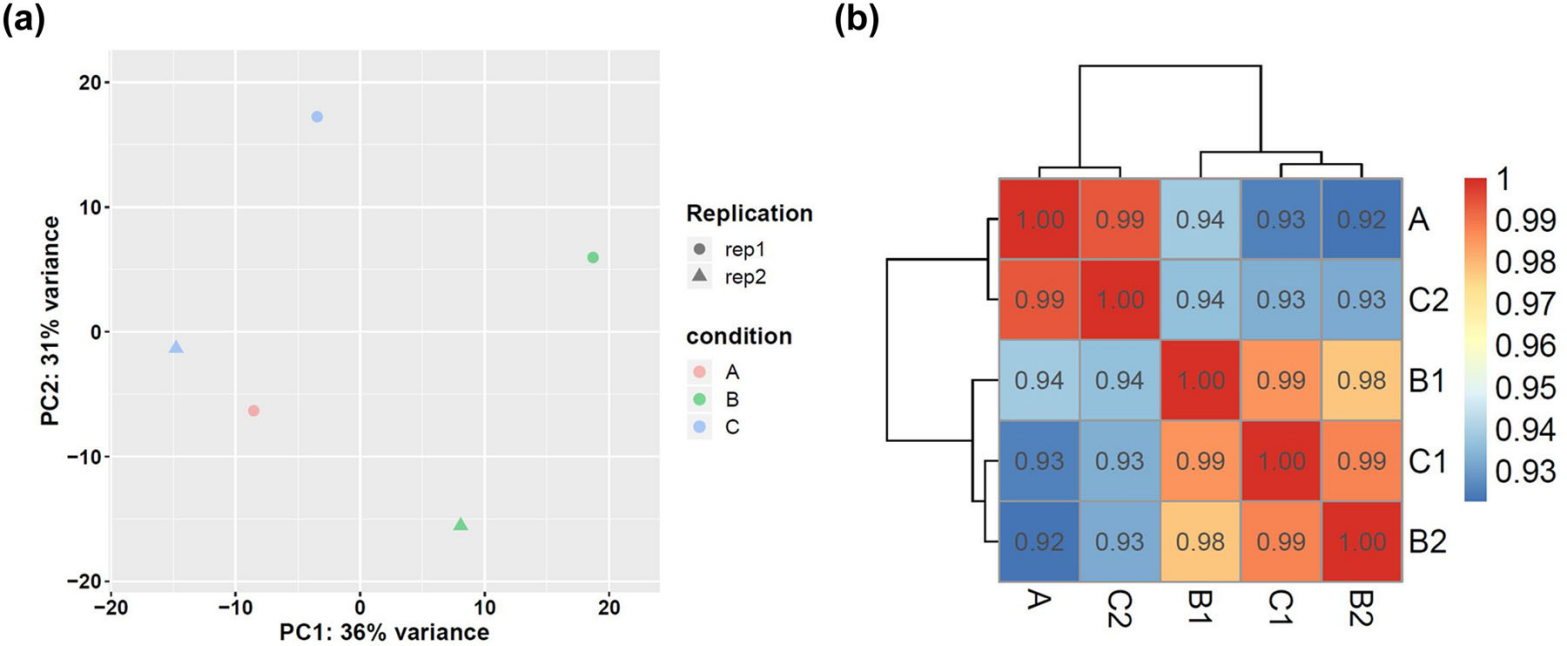
The variance and correlation of tested samples in this study are within the statistical range; (a) Principal component analysis diagram; (b) sample correlation matrix diagram

By collecting brain tissues of the three groups of rats and extracting total RNA, RNA sequencing analysis was performed to explore the network mechanism of EBI after PKR improved SAH. Figure **4** shows the plot of principal component analysis (PCA, Figure 4a) and correlation matrix of the tested samples (Figure 4b). After PCA and sample correlation analysis, the variance and correlation of the samples tested in this study were within a reasonable range.

The differential genes with |fold2change | ≥ 10 were selected for heatmap mapping. Genes differentially expressed in the CON group versus the SAH component may play a role in SAH pathogenesis, while genes differentially expressed between the SAH+C16 and SAH groups may play a role in suppressing PKR‐improved SAH. A total of 398 genes were downregulated and 42 genes were upregulated in the CON group compared to the SAH group. A total of 117 genes were upregulated in the SAH group compared to the SAH+C16 group. A total of 117 genes were downregulated and 442 genes were upregulated in the SAH group compared to the SAH+C16 group. The specific list of differentially expressed genes is shown in Table S1.

**FIGURE 5:**
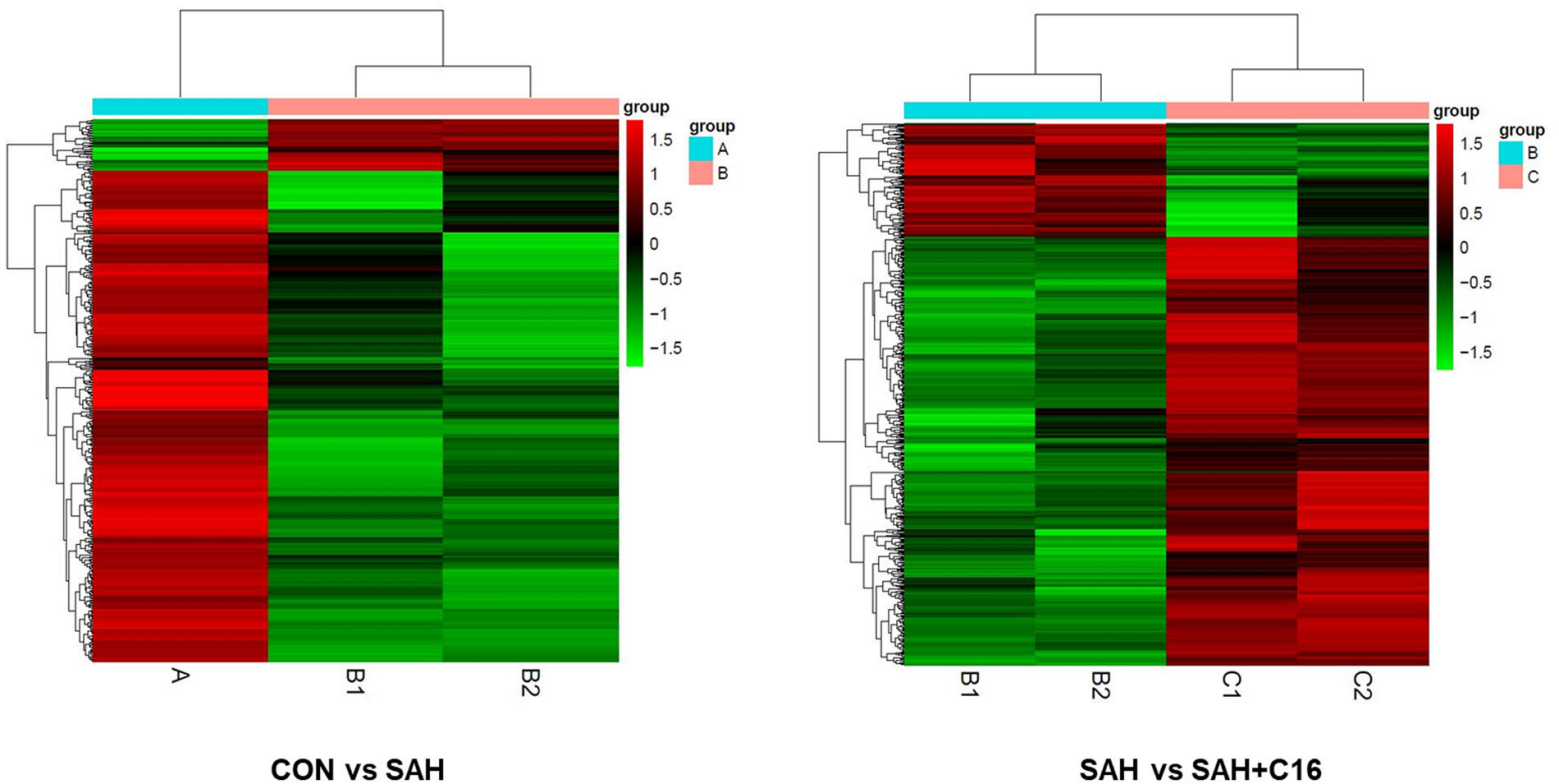
The heat maps of differentially expressed mRNAs

Figure **5** shows the heat map of differentially expressed mRNAs between different groupings. As the figure shows the two‐way clustering analysis with differentially expressed mRNAs versus groups, the shades of color represent the high or low mRNA expression, red represents high expression, and green represents low expression.

**FIGURE 6:**
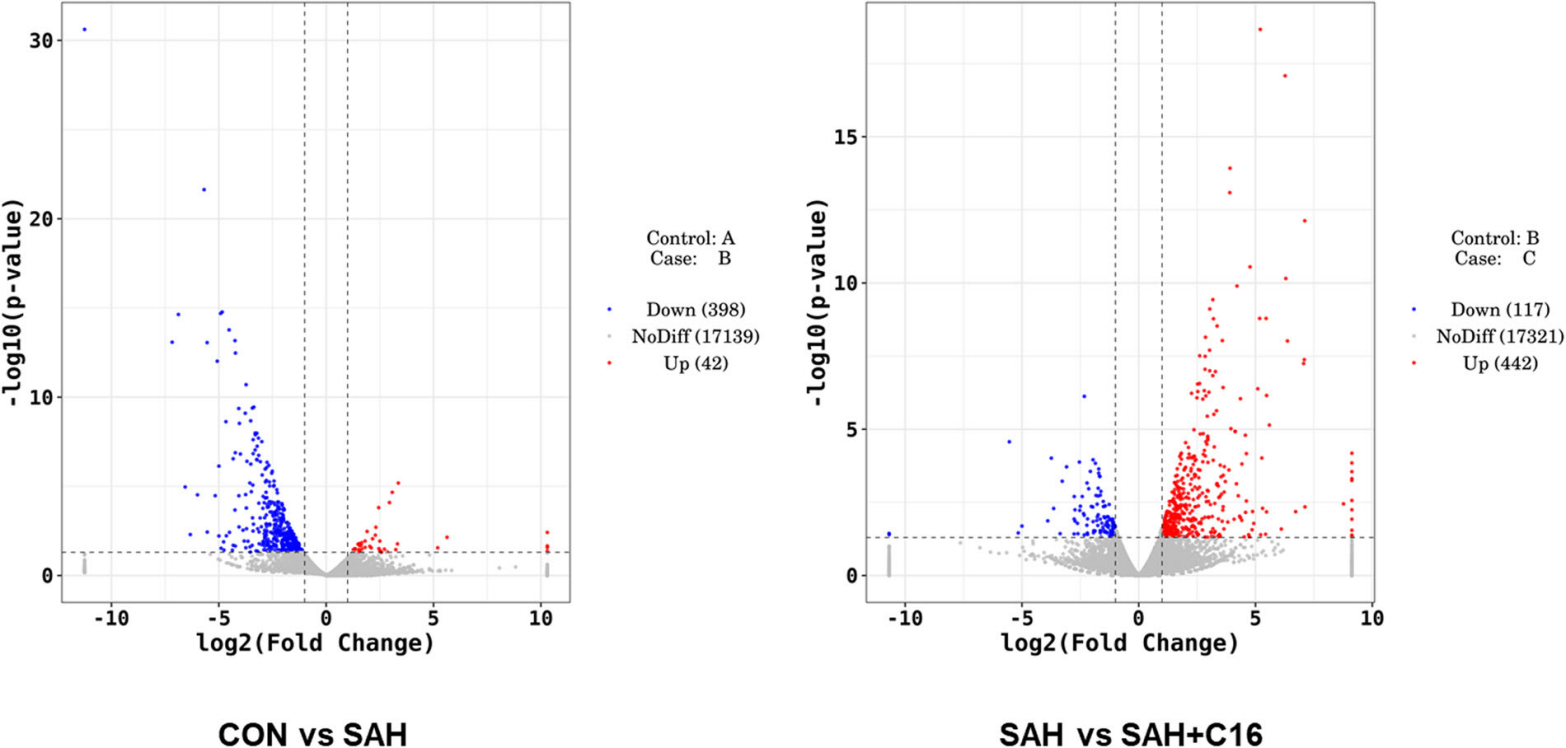
The volcano plots of differentially expressed mRNAs

Figure **6** shows the volcano plots of differentially expressed mRNAs between the groups. Each point in the graph represents an mRNA, and the horizontal coordinate indicates the log2 (fold change) value of the fold difference in expression of mRNA between the two groups of samples. The vertical coordinate indicates the negative log value of the *p* value of the change in gene expression. The larger the absolute value of the horizontal coordinate, the greater the expression fold difference between the two samples. The larger the value of the vertical coordinate, the more significant the differential expression.

**FIGURE 7:**
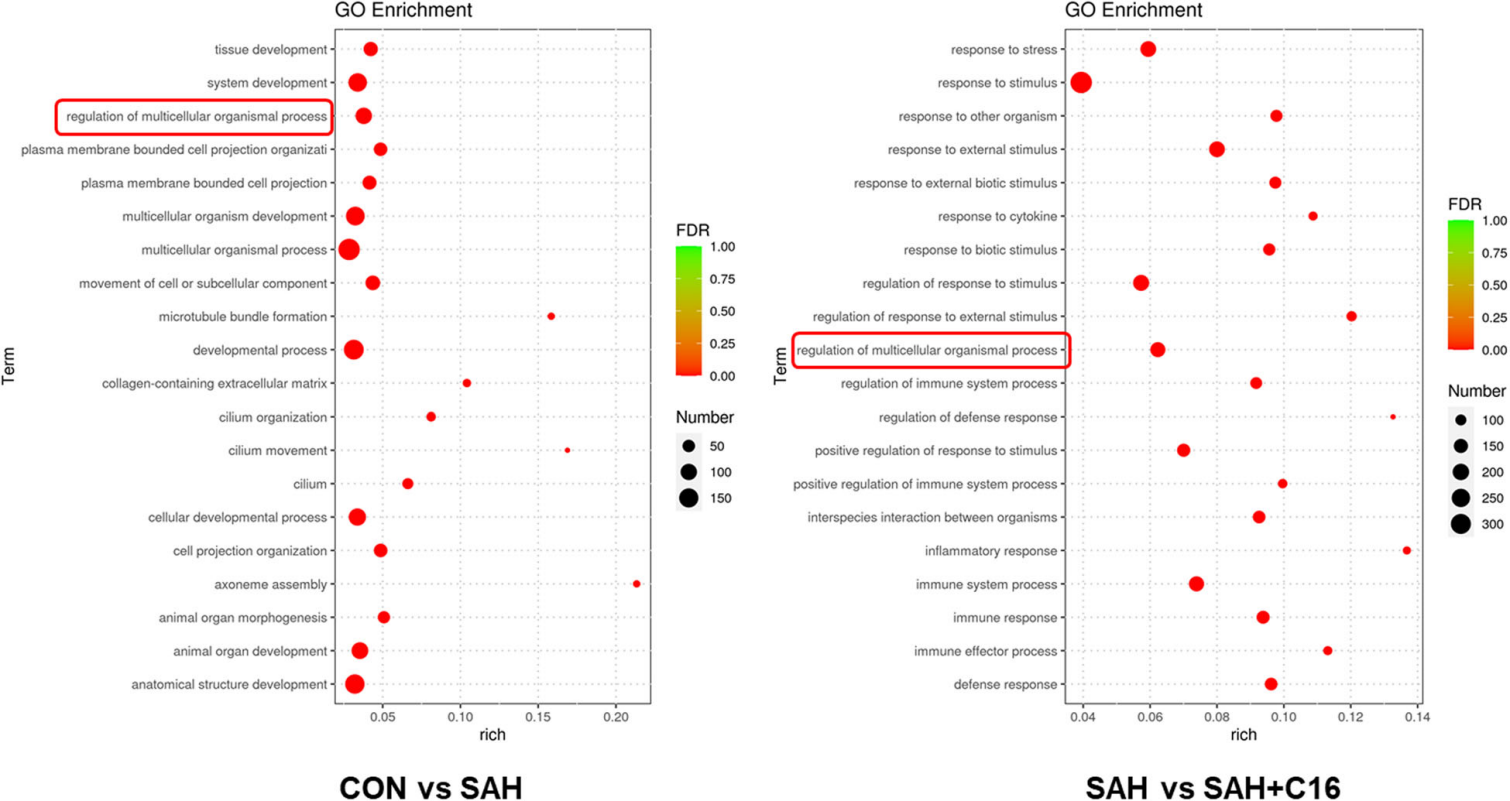
The GO enrichment analysis of differential mRNA target genes

Figure **7** shows the GO enrichment analysis of differential mRNA target genes, the horizontal coordinate is the GO classification, the secondary function of GO, the vertical coordinate is the percentage of target genes, and the number of target genes. The differentially expressed mRNAs in the SAH and CON groups were enriched in the multicellular organismal process, system development, developmental process, anatomical structure development, multicellular organism development, cellar development process, and animal organ development signaling pathway. It can be seen that the occurrence and development of SAH mainly depend on the organism development‐related pathways. The differentially expressed mRNAs in the SAH and SAH + C16 groups were enriched in the response to stimulus, response to stress, regulation of response to stimulus, and response to external stimulus signaling pathway. This suggests that the role of PKR in SAH is focused on stress response. It is noteworthy that the differential mRNA target genes GO analysis among the three groups were all enriched in the regulation of multicellular organismal process signaling pathway, suggesting the mechanism of PKR in the occurrence and development of SAH.

**FIGURE 8:**
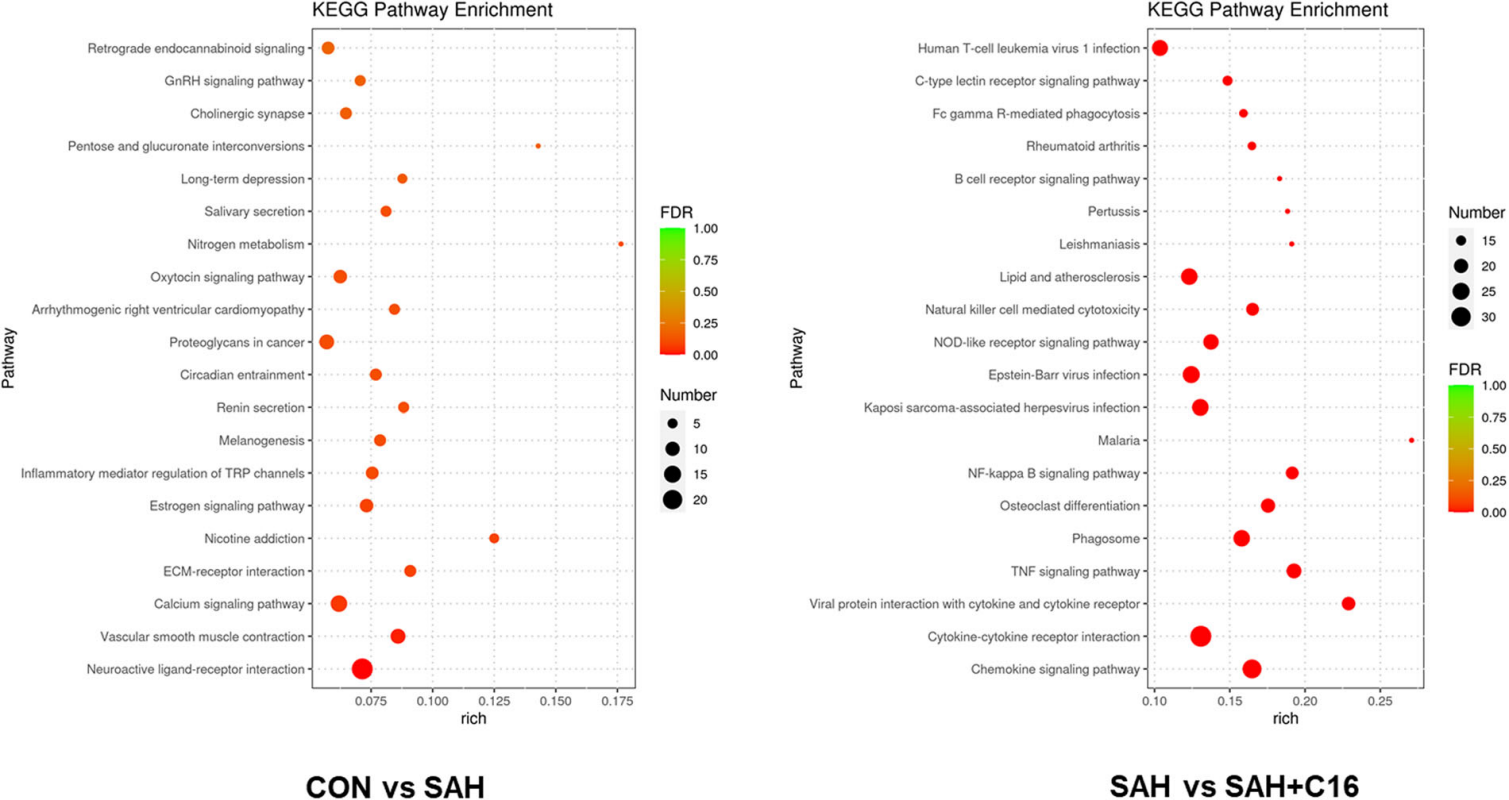
The KEEG enrichment analysis of differential mRNA target genes

**FIGURE 9:**
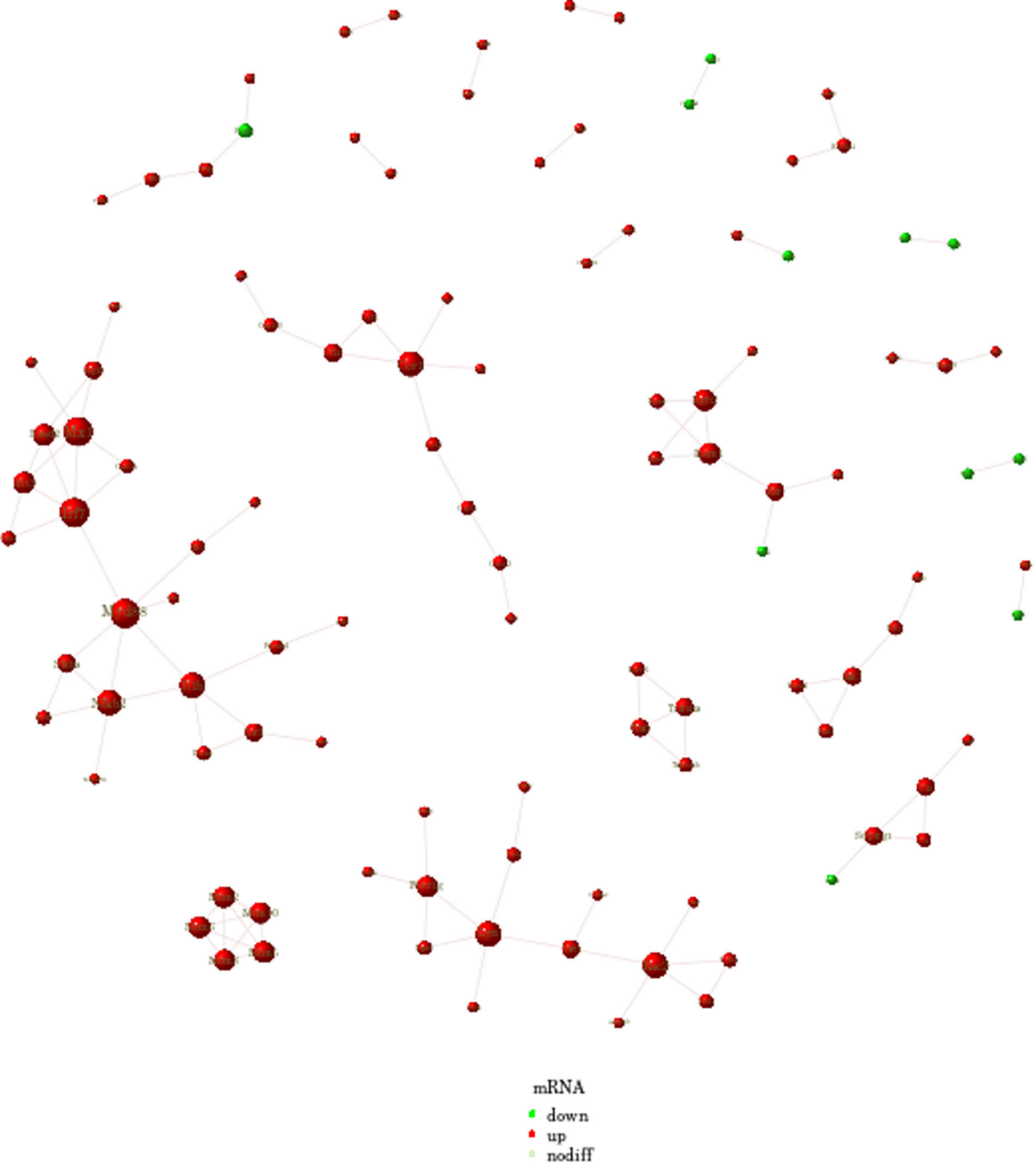
The interaction diagram of protein network of the differential mRNA target genes between inhibiting of PKR and SAH

Figure **8** shows the KEGG enrichment analysis of mRNA with differential expression. The horizontal coordinate is enrichment factor, that is, background genes of mRNA with differential expression in this pathway, the vertical coordinate is corresponding pathway description, the bubble size is the number of genes with differential expression, and the bubble color is the enrichment significance *p* value. The differentially expressed mRNAs in the SAH and CON groups were enriched in the neuroactive ligand–receptor interaction, calcium signaling pathway, proteoglycans in cancer, and retrograde endocannabinoid signaling pathways. The differentially expressed mRNAs in the SAH and SAH + C16 groups were enriched in the human T‐cell leukemia virus 1 infection, cytokine–cytokine receptor interaction, chemokine signaling pathway, lipid and atherosclerosis, and Epstein‐Barr virus infection. Figure **9** shows the reciprocal network of differentially expressed mRNA target proteins in the SAH group and the SAH‐C16 group.

## 4 DISCUSSION

EBI is a key factor in the poor prognosis of SAH. Ischemic brain injury has complex pathophysiological mechanism, and neuron injury is an important part of it (Park et al., 2018). Studies have identified the promotion of cellular ferroptosis by PKR in mouse hippocampal neurons (Hirata et al., 2019), and PKR has been found to be associated with neurodegenerative diseases such as Alzheimer and may be involved in a variety of cardiovascular diseases associated with chronic inflammation and is the most promising drug target. Therefore, in this study, we constructed a rat model of SAH with PKR inhibition, explored the specific mechanism of PKR involvement in EBI after SAH in rats by detecting ferroptosis‐related indicators, and analyzed the key signaling pathways of PKR involvement in SAH with the help of RNA‐seq to reveal the molecular mechanism of PKR‐mediated neuronal ferroptosis.

The molecular biochemical basis of neurodegenerative diseases such as Alzheimer's disease and Parkinson's disease is mitochondrial dysfunction, reduced GSH, and abnormal free iron metabolism (Dias et al., 2013; Schulz et al., 2000; Smeyne & Smeyne, 2013). Abnormal mitochondrial function, altered GSH, and free iron status may lead to oxidative stress and contribute to cascade neuronal cell death and progression of neuroprogressive diseases (Halliwell, 2006). Glutamate‐induced oxidative cell death in mouse hippocampal HT22 cells (Tan et al., 2001) and Erastin‐induced ferroptosis in certain cancer cells (Dixon et al., 2014) are both typical cellular models of endogenous oxidative stress, a nonapoptotic form of iron‐dependent cell death, and both cellular models cause the mitochondrial dysfunction, reduced GSH, and abnormal free iron metabolism.

The interrelationship between ferroptosis and endoplasmic reticulum stress is also a current research hotspot, where the activation of sustained endoplasmic reticulum stress instead activates the apoptotic program and thus induces apoptosis. The co‐occurrence of neuroinflammation and oxidative stress in ischemic brain injury also suggests a complex mechanism involved in neuronal injury (Alvi et al., 2020; Shah et al., 2019). Stockwell's group (Yang et al., 2014) found that treatment of HT1080 cells with Erastin or Sorafenib induced iron death and activated endoplasmic reticulum stress when they were treated with anticancer drugs targeting system XC. Some researchers have also suggested that endoplasmic reticulum stress may be the link between the interaction between ferroptosis and apoptosis (Hong et al., 2017). It has even been reported that endoplasmic reticulum stress promotes ferroptosis in certain disease conditions: Xu et al. (2020) found that ferroptosis is an important pathway causing colonic epithelial cell death in ulcerative colitis (UC), and that the endoplasmic reticulum stress marker molecule GRP78, as well as the PERK‐ATF4‐CHOP pathway, are important in the death of colonic epithelial cells in UC. The endoplasmic reticulum stress marker GRP78 and the PERK‐ATF4‐CHOP pathway were significantly activated in the colonic epithelial cells of UC mice. Treatment with GSK414, an inhibitor of PERK, significantly inhibited DSS‐induced iron death and significantly decreased the iron levels and lipid peroxidation levels in the colonic epithelial cells of mice.

In our group, we found that the endoplasmic reticulum stress PERK‐ATF4 pathway upregulates the expression of HSPA5, a member of the heat kinin family, which binds to GPX4 to form a complex that inhibits its degradation and thus prevents ferroptosis from occurring. The PERK (PKR‐like reticulum kinase) signaling pathway is one of the three signaling pathways that are activated in response to external stress and are involved in various physiological and biochemical processes in the cell (Bu & Diehl, 2016). It has been reported that NF‐E2‐related factor 2 (NRF2), a gene downstream of PERK, is also activated upon PERK activation (Cullinan et al., 2003). Furthermore, recent studies have confirmed that NRF2 is a negative regulator gene of ferroptosis (Dodson et al., 2019; Fan et al., 2017).

PKR kinases are associated with ferroptosis, and unlike the inhibitory effect of the PERK‐ATF4 pathway on iron death, the role of PKR is to promote the occurrence of iron death (Hirata et al., 2019). Although the mechanisms by which PKR regulates ferroptosis have been investigated, the reports that have been done are limited to the regulation of phosphorylation of individual kinases, and it is not clear whether these kinases are key genes downstream of PKR. SAH puts neuronal cells in a state of intense mechanical stress, oxidative stress, and endoplasmic reticulum stress (Zhang et al., 2018), and ferroptosis is one of the most important forms of cell death in SAH. In view of this, in this study, RNA sequencing analysis was performed on brain tissue from SAH and C16‐treated SAH rats. The differentially expressed mRNAs were analyzed by GO enrichment and revealed that the target genes were mainly concentrated in stress response‐related pathways, which corresponded to the role played by PKR in endoplasmic reticulum stress.

In conclusion, our study revealed that inhibition of PKR may significantly ameliorate SAH‐induced EBI by regulating iron death, and the mechanism may be associated with endoplasmic reticulum stress by RNA sequencing analysis.

## CONFLICT OF INTEREST

The authors declare there is no conflict of interest.

### PEER REVIEW

The peer review history for this article is available https://publons.com/publon/10.1002/brb3.2722.

## Supporting information

Supplementary information

## Data Availability

The datasets generated and analyzed during the current study are available from the corresponding author on reasonable request.
